# A Comparative Study of Gene Expression in Menstrual Blood-Derived Stromal Cells between Endometriosis and Healthy Women

**DOI:** 10.1155/2022/7053521

**Published:** 2022-01-11

**Authors:** Seyedeh Saeideh Sahraei, Faezeh Davoodi Asl, Naser Kalhor, Mohsen Sheykhhasan, Hoda Fazaeli, Sanaz Soleymani Moud, Azar Sheikholeslami

**Affiliations:** ^1^Department of Reproductive Biology, Academic Center for Education, Culture, and Research (ACECR), Qom Branch, Qom, Iran; ^2^Department of Mesenchymal Stem Cells, Academic Center for Education, Culture, and Research (ACECR), Qom Branch, Qom, Iran; ^3^Midwifery Ward, Infertility Treatment Center, Academic Center for Education, Culture, and Research (ACECR), Qom Branch, Qom, Iran

## Abstract

**Background:**

Research into the pathogenesis of endometriosis would substantially promote its effective treatment and early diagnosis. Currently, accumulating evidence has shed light on the importance of endometrial stem cells within the menstrual blood which are involved in the establishment and progression of endometriotic lesions in a retrograde manner.

**Objectives:**

We aimed to identify the differences in some genes' expression between menstrual blood-derived mesenchymal stem cells (MenSCs) isolated from endometriosis patients (E-MenSCs) and MenSCs from healthy women (NE-MenSCs).

**Methods:**

Menstrual blood samples (2-3 mL) from healthy and endometriosis women in the age range of 22–35 years were collected. Isolated MenSCs by the Ficoll-Paque density-gradient centrifugation method were characterized by flow cytometry. MenSCs were evaluated for key related endometriosis genes by real-time-PCR.

**Results:**

E-MenSCs were morphologically different from NE-MenSCs and showed, respectively, higher and lower expression of CD10 and CD9. Furthermore, E-MenSCs had higher expression of Cyclin D1 (a cell cycle-related gene) and MMP-2 and MMP-9 (migration- and invasion-related genes) genes compared with NE-MenSCs. Despite higher cell proliferation in E-MenSCs, the BAX/BCL-2 ratio was significantly lower in E-MenSCs compared to NE-MenSCs. Also, the level of inflammatory genes such as IL1*β*, IL6, IL8, and NF-*κ*B and stemness genes including SOX2 and SALL4 was increased in E-MenSCs compared with NE-MenSCs. Further, VEGF, as a potent angiogenic factor, showed a significant increase in E-MenSCs rather than NE-MenSCs. However, NE-MenSCs showed increased ER-*α* and *β*-catenin when compared with E-MenSCs.

**Conclusion:**

Here, we showed that there are gene expression differences between E-MenSCs and NE-MenSCs. These findings propose that MenSCs could play key role in the pathogenesis of endometriosis and further support the menstrual blood retrograde theory of endometriosis formation. This could be of great importance in exploiting promising therapeutic targets and new biomarkers for endometriosis treatment and prognosis.

## 1. Introduction

Endometriosis is a benign inflammatory disease in women caused by the outgrowth of endometrial tissue outside the uterus [1, 2]. The exact cause of endometriosis pathogenesis has not been determined yet [3, 4]. However, the most important accepted cause of endometriosis is the retrograde of menstrual blood flow into the pelvic cavity and the settling of menstrual blood cells outside the uterine cavity [5]. Despite being a benign disease, endometriosis needs to be evaluated and treated due to the complications it makes for women, such as infertility and severe pelvic pains. Chronic pain is extremely common in this disease, and almost 40% of patients with endometriosis suffer from infertility problems, whereas conventional treatments including hormone therapy and surgery are not very effective [6, 7].

Evidence of studies showed that there are stem cells with high proliferation and differentiation potential in menstrual blood called menstrual blood-derived stem cells (MenSCs). These cells not only are morphologically and functionally similar to cells directly isolated from the endometrium but also express both markers of mesenchymal and embryonic stem cells, such as Oct-4, SSEA-4, Nanog, c-kit (CD117), CD44, CD90, and CD105, as well [8–11].

It has been shown that molecular signaling pathways and expression profile of some genes in the MenSCs of women with endometriosis are different from healthy women, in which awareness of them can be a step towards diagnosing the pathogenesis and providing effective treatment strategies for this disease [4, 12]. Analyzing the molecular network of endometriosis revealed that the genes and the signaling pathways that play important roles in the establishment and spread of this disease are including inflammatory genes, such as IL-1*β*, Cox-2, NF-*κ*b, HIF-1*α*, TNF-*α*, IL-6, and IL-8; the stemness and pluripotency-related genes such as SOX2, OCT4, SALL4, and Nanog; the genes related to steroidogenic pathway such as ER; the apoptosis related genes such as Bcl-2 and BAX; the genes of angiogenesis, such as VEGF; the genes involved in tissue breakdown which then facilitates migration and invasion, such as MMP-2 and MMP-9; the cell cycle-related genes, such as cyclin D1; and the Wnt/*β*catenin signaling pathway [13–15].

Inflammation acts as a key pathway in the pathogenesis of endometriosis [16]. So, the release of several cytokines related to inflammation, including IL-1*β*, Cox-2, NF-*κ*b, HIF-1*α*, TNF-*α*, IL-6, and IL-8, could modulate proliferation and vascularization followed by endometriosis development [16]. In addition, it was reported that inflammation pathways and inflammasome may be modulated by sex steroid hormones in endometriosis [17].

Also, the stemness and pluripotency-related genes play an important role in the pathogenesis of endometriosis [15]. Several research studies have reported that the expression of stemness genes such as OCT-4 and SOX-2 was increased in patients with endometriosis [15, 18, 19]. It seems that increased expression of these genes may contributes to the development and spread of endometriosis [15].

Wnt/*β*-catenin signaling can also be considered as an important pathway for endometriosis development [20]. It was found that endometriosis could be affected through estrogen and progesterone modulation by regulating Wnt/*β*-catenin signaling [20]. Furthermore, the cyclical changes in proliferation and differentiation of endometrial cells are modulated by estrogen and progesterone via regulating Wnt/*β*-catenin signaling [21].

Furthermore, angiogenesis may be considered as one of the steps in the development of endometriosis [22]. VEGF as one of the most important and potent angiogenic factors may be involved in the spread of endometriosis by affecting angiogenesis [22]. Moreover, endometriotic cells may produce and release VEGF and may cause angiogenesis in this way [23]. So, some blood supply is needed for growth and increased some VEGF was observed in endometriosis.

Also, it was demonstrated that the expression of MMP-2 and MMP-9 is overexpressed in the eutopic endometrium as well as the endometriotic lesions of patients with endometriosis [24, 25]. It was identified that upregulating MMP-2 and MMP-9 could participate in tissue breakdown which then induces migration and invasion [26, 27].

In addition, apoptosis can be regarded as a critical process in the endometriosis pathogenesis [28]. It was confirmed that endometriosis-derived eutopic endometrium shows overexpression of antiapoptotic factors and downregulated expression of proapoptotic factors compared with healthy donor-derived endometrium [28].

In this study, in order to better understand the affected molecular pathways involved in the endometriosis process, we planned to investigate and compare the gene expression patterns of MenSCs obtained from patients with endometriosis and healthy women.

## 2. Materials and Methods

### 2.1. Human Specimens

This experimental study was approved by the ethics committee of Islamic Azad University, Qom branch (IR.IAU.QOM.REC.1399.064). All patients signed a written informed consent before participating in the study. Nonendometriosis women were considered as the control group (*n* = 3), while women with stages III-IV endometriosis were allocated to the patient group (*n* = 3). All the individuals in control and patient groups were undergoing laparoscopy for infertility or pelvic pain, respectively. The following inclusion criteria were observed to enroll participants: age range of 25 to 35 years, body mass index (BMI) of 18–28 kg/m^2^ in both groups, and no hormonal treatments for at least the last 3 months. Moreover, in the case of the patient group whose deep endometriosis was confirmed by transvaginal ultrasound and magnetic resonance imaging, having ovulatory cycles with irregular menstrual periods in endometriosis cases, no previous surgery for endometriosis women, and no history of malignancies or autoimmune diseases should be observed.

### 2.2. Isolation and Culture of MenSCs

During the second or third day of menstruation, at least 2 mL of menstrual blood was collected by a Pipelle endometrial sampling catheter and subsequently transferred to the laboratory after 3 appropriate cases in each group were selected. At first, EDTA 0.5 mM was added to the blood sample, and then, an equal volume of blood sample was carefully added to Ficoll-Paque media (Lymphodex, innotrain, Germany) and centrifuged at 600 × g for 30 min at room temperature. Plasma and platelets in the upper layer were eliminated during density gradient centrifugation, leaving the mononuclear cell layer intact at the interface. After transferring mononuclear cell layer to the sterile centrifuging tube, it was washed twice with phosphate buffer saline (PBS). Cell pellets were seeded with Dulbecco's modified Eagle's low glucose (DMEM-LG) medium supplemented with 10% FBS (Gibco, Grand Island, USA) and 1% penicillin/streptomycin (Gibco, Grand Island, USA) and incubated at 37°C with 97% humidity and 5% CO_2_. For all the experiments, approximately 5 × 10^5^ cells at their 3^rd^ passage were used in each group. The endometriosis and healthy women's isolated MenSCs are referred to as E-MenSCs and NE-MenSCs, respectively.

### 2.3. Flow Cytometry for MenSC Markers

The expression level of positive (CD29, CD90, CD105, CD44, CD73, and CD10) and negative (CD34, CD45, CD133, and CD38) cell surface markers was investigated to confirm the isolated cells as MenSCs. FITC-conjugated monoclonal antibodies against CD34 and CD133 as well as PE-conjugated monoclonal antibodies for CD10, CD44, CD45, and CD73 were purchased from BD Biosciences (San Jose, CA, USA). PE-conjugated anti-CD105, CD90, CD29, and CD38 were from R&D Systems (Minneapolis, MN, USA). Flow cytometry was performed using an FC500 flow cytometer (Beckman Coulter, Fullerton, CA), and Beckman Coulter CXP software was used to analyze data. In this procedure, all of the antibodies were employed at the amounts advised by the manufacturers.

### 2.4. Real-Time Polymerase Chain Reaction (PCR) and Gene Expression Analysis

Total RNA was extracted from both E-MenSCs and NE-MenSCs using “Gene All Kit (Gene All Biotechnology, Seoul, Korea) according to the manufacturer's instructions. The Nanodrop 2000 spectrophotometer (Thermo Fisher Scientific, Wilmington, USA) was used to evaluate RNA purity and quantity at 260/280 nm. The single-strand cDNA was synthesized via reverse transcription using a transcription kit (Yekta tajhiz, Iran).” To assess the level of selected gene expression, quantitative real-time PCR tests were performed in triplicate (Table 1). All the primers were purchased from Pishgam Biotech, Iran. The glyceraldehyde-3-phosphate dehydrogenase (GAPDH) gene was selected as an internal reference for standardizing gene expression levels. The fold change of mRNA expressions for target genes was calculated using the 2^-∆∆Ct^ technique. Real-time PCR was performed according to the manufacturer's instructions using RealQ Plus Master Mix Green (AMPLIQONIII). In brief, 10 *μ*L SYBR green mix, 1 *μ*L cDNA (250 ng), and 1 *μ*L of each PCR forward and reverse primers in 5 pmol *μ*L^−1^ were mixed together. Millipore water was added to achieve the final volume of 20 *μ*L.

The primer sequences are listed in Table 1. The threshold cycle (CT) was determined manually for each run. Relative mRNA level was expressed as the relative fold change and calculated using the formula 2^–^∆∆^CT^ = 2^–^(∆^CT(sample)−^∆^CT(calibrator)^), where each ∆CT = ∆CT target–∆CT GAPDH. The quantification of mRNA was performed as a value relative to an internal reference for *GAPDH*.

### 2.5. Statistical Analysis

The experimental data are presented as the mean ± standard deviation (SD) and compared using ANOVA. The statistical significance was determined using an ANOVA with a multiple comparison test, followed by the Tukey test. The threshold for statistical significance was established at *P* ≤ 0.05. For technical and biological repeatability, all experiments were carried out in triplicate.

## 3. Results

### 3.1. Identification of MenSCs

**Figure 1:**
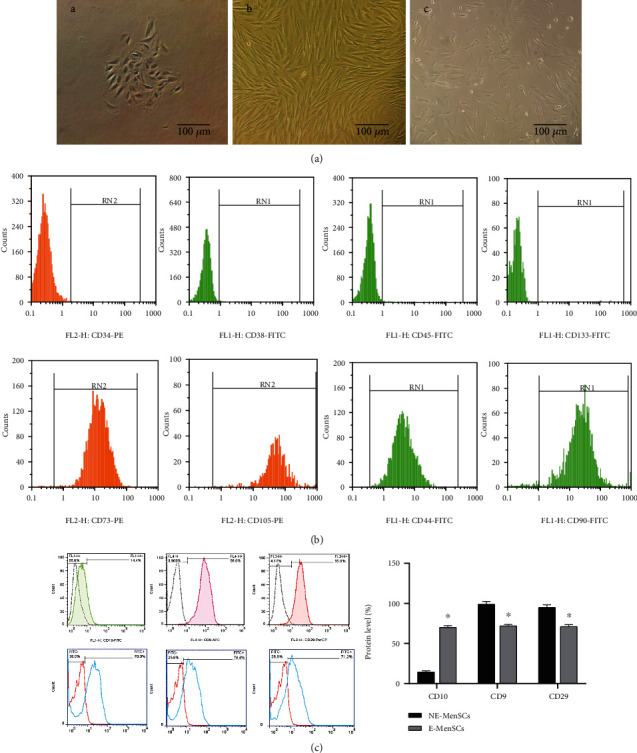
(a) Typical characteristics of MenSCs. (A, B) Morphology of EnSCs. P0 and P3 NE-MenSCs showed a typical spindle-shaped, polygonal, swirling, and fibroblast-like morphology (C) but E-MenSCs showed an irregular morphology less stretched and more circular (×100 magnification); (b) The expression of MenSC surface markers was detected by flow cytometry so that they were positive for CD90, CD44, CD29, CD73, and CD105, while negative for CD34, CD133, CD45, and CD38; (c) compare CD10, CD9, and CD29 marker in E-MenSCs with NE-MenSCs.

During primary culture, MenSCs exhibited a colony-like morphology, which was clearly observed in both NE-MenSCs and E-MenSCs (Figure 1(a), A, B, and C). As previously reported [29], subcultured NE-MenSCs demonstrated growth characteristics typical of a spindle-shaped, fibroblast-like morphology with a radial or helical growth pattern; however, subcultured E-MenSCs exhibited an irregular morphology different from NE-MenSCs. Flow cytometric analysis of passage 3 MenSCs demonstrated that both NE-MenSCs and E-MenSCs were positive for CD44, CD73, CD90, CD105, CD29, CD9, and CD10 expression but negative for CD34, CD45, CD133, and CD38 expression (Figure 1(b)). To compare E-MenSCs and NE-MenSCs concerning the expression level of CD markers, we used the mean fluorescence intensity (MFI). In our results, CD10 expression was significantly higher in E-MenSCs compared with NE-MenSCs, which can be considered as a useful marker in the diagnosis of endometriosis. In contrast, CD9 and CD29 expression was significantly lower in E-MenSCs compared with NE-MenSCs (Figure 1(c)).

### 3.2. qRT-PCR Analysis

Based on the previous studies [9, 30], the endometrial stem cells of women with endometriosis are different from those of healthy women, which can be a step towards diagnosing the pathogenesis and providing effective treatment strategies for this disease [4].

#### 3.2.1. Expression of Stemness Genes in E-MenSCs

**Figure 2:**
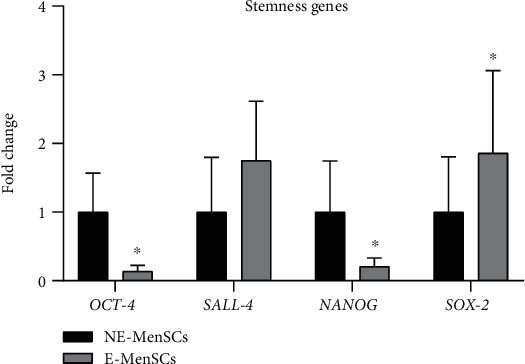
Quantitative assessment of OCT-4, SALL-4, NANOG, and SOX-2 gene expression. Gene expression in both MenSCs types was evaluated by qRT-PCR. SOX-2 and SALL-4 genes showed an upregulation in E-MenSCs compared with that in NE-MenSCs, while OCT-4 and NANOG gene expression was lower in E-MenSCs. ^∗^*P* ≤ 0.05.

Evaluation of stemness-related genes showed that mRNA expression of *SOX-2* was significantly increased in E-MenSCs (1.8574 fold, *P* ≤ 0.04) compared with NE-MenSCs. In contrast, the expression of *OCT-4* and *NANOG* was lower in E-MenSCs compared with NE-MenSCs (*P* =0.0). There was also no significant change in the level of *SALL-4* (1.751187 fold, *P* ≤ 0.06) gene expression in endometriosis cells (Figure 2).

#### 3.2.2. Expression of Apoptosis Genes in E-MenSCs

**Figure 3:**
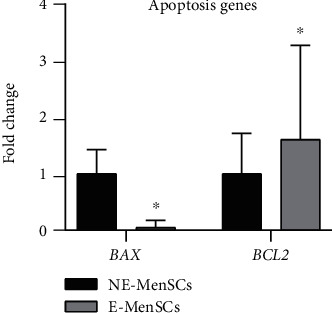
Quantitative assessment of BAX and BCL-2 gene expression. Gene expression in both MenSC types was evaluated by qRT-PCR. BAX and BCL-2 genes showed an upregulation in E-MenSCs compared with that in NE-MenSCs. ^∗^*P* ≤ 0.05.

Evaluation of apoptosis-related genes showed that E-MenSCs significantly decreased mRNA expression of BAX (proapoptotic gene) (0.052 fold, *P* = 0.05) and significantly increase mRNA expression of BCL-2 (antiapoptotic gene) (1.60 fold, *P* = 0.01) in endometriosis cells, as compared with NE-MenSCs (Figure 3). The balance between pro- and antiapoptotic members of this family can determine the cellular fate. Moreover, the BAX/BCL-2 ratio was significantly lower (0.06) in E-MenSCs compared to NE-MenSCs.

#### 3.2.3. Migration and Invasion in E-MenSCs

**Figure 4:**
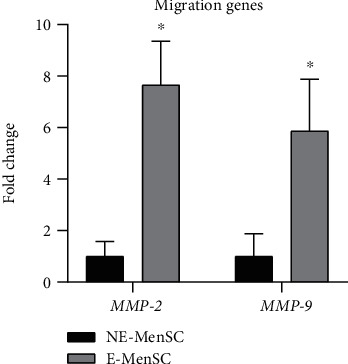
Quantitative assessment of MMP-2 and MMP-9 gene expression. Gene expression in both MenSC types was evaluated by qRT-PCR. MMP-2 and MMP-9 genes showed an upregulation in E-MenSCs compared with that in NE-MenSCs. ^∗^*P* ≤ 0.05.

To investigate the migration and invasion of endometriotic stem cells, we evaluated mRNA expression of MMP-2 and MMP-9 genes. Our data showed a higher expression of MMP-2 and MMP-9 genes in endometriosis cells (E-MenSCs) (7.6 and 5.8 fold), as compared with NE-MenSCs (Figure 4). Our results confirmed the previous findings, demonstrating the superior migratory capacity of E-MenSCs.

### 3.3. Expression of Inflammatory Genes in E-MenSCs

**Figure 5:**
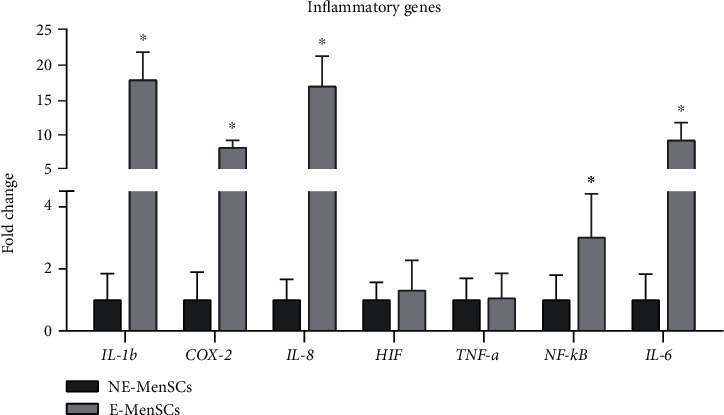
Quantitative assessment IL-1b, COX-2, IL-8, HIF, TNF-a, NF-*κ*B, and IL-6 gene expression. Gene expression in both MenSC types was evaluated by qRT-PCR. IL-1b, IL-8, NF-*κ*B, and IL-6 genes showed an upregulation in E-MenSCs compared with that in NE-MenSCs, while COX-2, HIF, and TNF-a gene expression was similar to the control group. ^∗^*P* ≤ 0.05.

We assessed the expression of several key inflammatory genes which are expressed at high or moderate levels in MenSCs (Figure 5). Compared with NE-MenSCs, E-MenSCs expressed greater IL-1*β* (17.69, *P* < 001), IL-6 (9.063, *P* < 001), IL-8 (16.795 fold, *P* < 001), NF-*κ*B (3.017 fold, *P* < 001), and COX-2 (8.033 fold, *P* < 005), while in E-MenSCs, HIF (1.304 fold, *P* <001) and TNF-*α* (1.061914 fold, *P* < 001) genes had similar expression levels to NE-MenSCs. Our results show that inflammatory genes are strongly influential in endometriosis disease.

### 3.4. Expression of Mitotic Factor in E-MenSCs

**Figure 6:**
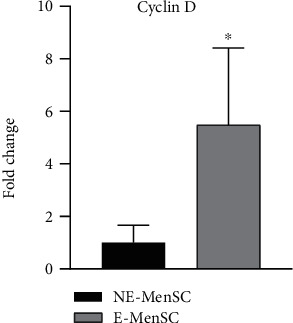
Quantitative assessment of Cyclin D gene expression. Gene expression in both MenSC types was evaluated by qRT-PCR. Cyclin D gene showed an upregulation in E-MenSCs compared with that in NE-MenSCs. ^∗^*P* ≤ 0.05.

Cyclin D1, as a mitotic cyclin, plays an integral role in many types of cancer. We observed cyclin D1 levels deregulated in endometriosis cell line which was significantly elevated (5.4 folds) in E-MenSCs (*P* < 0.01) compared with NE-MenSCs (Figure 6).

### 3.5. Expression of Angiogenic Factor in E-MenSCs

**Figure 7:**
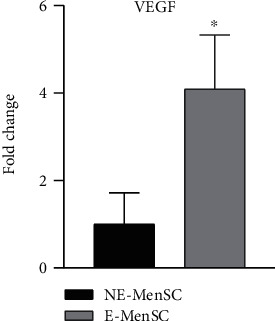
Quantitative assessment of VEGF gene expression. Gene expression in both MenSC types was evaluated by qRT-PCR. VEGF gene showed an upregulation in E-MenSCs compared with that in NE-MenSCs. ^∗^*P* ≤ 0.05.

As one of the most potent angiogenic factors, VEGF is postulated to be involved in the progress of ectopic lesions in endometriosis. Compared with NE-MenSCs, VEGF (proangiogenic factor) was expressed at high level in E-MenSCs, 4.09 fold (*P* < 0.05) (Figure 7).

### 3.6. Expression of Estrogen Receptor in E-MenSCs

**Figure 8:**
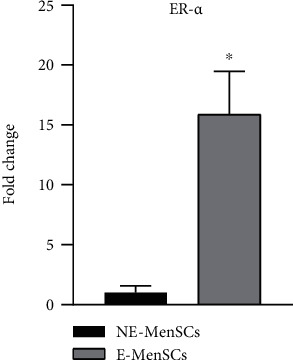
Quantitative assessment of ER-*α* gene expression. Gene expression in both MenSC types was evaluated by qRT-PCR. ER gene showed an upregulation in E-MenSCs compared with that in NE-MenSCs. ^∗^*P* ≤ 0.05.

ER expression may serve as a prognostic biomarker of aggressive endometriosis. We observed that ER-*α* gene was remarkably increased in E-MenSCs compared with NE-MenSCs (15.88 fold, *P* < 0.01) (Figure 8).

### 3.7. Wnt\*β* Catenin Signaling

**Figure 9:**
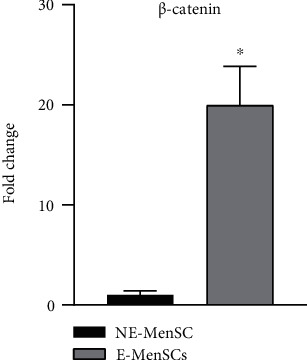
Quantitative assessment of *β*-catenin gene expression. Gene expression in both MenSC types was evaluated by qRT-PCR. The *β*-Catenin gene showed an upregulation in E-MenSCs compared with that in NE-MenSCs. ^∗^*P* ≤ 0.05.

*β*-Catenin is a dual-function protein, involved in regulation and coordination of cell-cell adhesion and gene transcription. *β*-Catenin also is involved in epithelial to mesenchymal transition and cell division. Our data revealed that E-MenSCs expressed greater *β*-catenin gene (19.97 fold, *P* < 0.05) (Figure 9).

## 4. Discussion

Endometriosis is a disease with a complex and multifactorial etiology. Recently, several reports have shown that endometrial stem cells are responsible for the endometriosis generation if shed in a retrograde manner in the menstrual cycle [31]. These menstrual blood-derived mesenchymal stem cells are thought to be involved in the formation of endometriosis [4]. In the present study, we analyzed the differences between E-MenSCs and NE-MenSCs in terms of morphology, expression level of some surface markers, expression of some key genes related to inflammation, apoptosis, angiogenesis, cell cycle control, adhesion, and steroidogenic pathways. In our results, E-MenSCs revealed a higher expression level of CD10 and lower expression of CD9 marker when compared with NE-MenSCs. CD10 could be considered as a useful marker in the diagnosis of endometriosis [32, 33]. In this regard, stromal cells existing in the milieu of several tumor types have been reported to differ from their normal counterparts with respect to the expression of various CD markers. Several studies have addressed the relationship between cancers and CD10 expression, demonstrating CD10 upregulation on tumor and stromal cells of different cancers such as breast and bladder [32–34].

On the other hand, the endometrial stromal cell expression of CD9 has been reported to be associated with infertility related to the endometrium [35]. CD9 is known as a marker shown to be associated with the implantation [36]. CD9 is associated with integrin adhesion receptors and controls integrin-dependent cell migration and invasion during blastocyst implantation [35]. CD9-deficient endometrium in mice failed to implant compared to the CD9-positive endometrium. CD9 is associated with blastocyst implantation by producing MMP-2 [37]. Overall, similar to other studies, higher expression of CD10 and lower expression of CD9 in E-MenSCs were observed in the current study.

### 4.1. Enhanced Stemness Gene Expression in E-MenSCs

In the present study, the expression level of some stemness-related genes was evaluated. NANOG and OCT4 had significant lower expression, whereas SOX2 gene showed significant increase in E-MenSCs compared to NE-MenSCs. Götte et al. in 2011 revealed a substantial increase in SOX2 expression in endometriosis rather than infertile patients without endometriosis [38]. Some studies have indicated increased expression of stemness-related markers in endometriotic tissue which can promote cell survival and self-renewal [39]. In 2016, Proestling et al. reported that endometriotic tissues have abnormal expression of SOX2, NANOG, and OCT4, in which OCT4 could stimulate endometrial cell migration [19]. We have also observed changes in the expression level of stemness genes which show contradictory results overall [18, 19].

### 4.2. Enhanced Apoptosis in E-MenSCs

The apoptosis rate in endometrial cells of endometriosis women was found to be slightly lower, suggesting that the survival rate of cells that reach the peritoneal cavity is higher in people with progressive endometriosis. Dmowski et al. analyzed the apoptotic index according to the stage of endometriosis and found that there was a trend towards decreased apoptosis with increasing stage of the disease, but the difference lacked statistical significance [40].

In 2020, Delbandi et al. analyzed the apoptosis in endometriosis by modulating Bcl-2 expression. In many studies, there is an inverse relationship between the level of apoptosis and the stage of endometriosis in apoptosis, and in general, there is an increase in angiogenesis and a decrease in apoptosis in endometriosis patients [41]. Actually, the ratio of Bax/Bcl-2 has an important involvement in apoptosis level. The Bax/Bcl-2 ratio was significantly lower in E-MenSCs compared to NE-MenSCs. Our data based on the Bax/Bcl-2 ratio confirm the decreased apoptosis in endometriosis stromal cells.

### 4.3. Enhanced Migratory and Angiogenic Capacities of E-MenSCs

Angiogenesis has an essential role in the establishment and growth of endometriotic lesions, regardless of apoptosis [42, 43]. Based on previous studies, it is shown that patients with endometriosis have much more active types of MMP-9 in epithelial cells and menstrual stroma than those without endometriosis [44]. Our results in consistent with the findings of the above study confirmed the increase in migration genes (MMP2 and MMP9) of E-MenSCs compared to NE-MenSCs. Furthermore, one of the most active angiogenesis factors, VEGF, plays an important role in both physiological and pathological angiogenesis [45]. Yerlikaya et al. in 2016 showed that there is an increase in angiogenesis and a decrease in apoptosis in endometriosis patients in general [45], which is in line with the results of our study. Growth factors, hormones, cytokines, and hypoxia stimulate VEGF development, and ectopic endometrium and peritoneal macrophages are sources of this factor in endometriosis [41].

### 4.4. Immunomodulatory Dysfunction of E-MenSCs

In endometriosis, local proinflammatory mediators such as interleukin (IL)-1b and tumor necrosis factor- (TNF-) a stimulate the nuclear factor kB (NF-*κ*B) and hypoxia inducible factor- (HIF-) 1a signaling pathways, increasing COX-2 expression [46]. COX-2 is an inducible enzyme that is normally absent in physiologic conditions but is rapidly released in pathological conditions such as endometriosis after stimulation by cytokines and proinflammatory agents. COX-2 expression in peritoneal macrophages was substantially increased in women with endometriosis [47]. In the present research, consistent with previous studies, a significant increase in the expression of inflammatory genes such as IL-1*β*, IL-6, IL-8, COX-2, and NFkB was observed in E-MenSCs compared with NE-MenSCs. Zhang and colleagues in 2018 showed that inflammation and estrogen form a positive feedback loop in ectopic endometriotic lesions, increasing the expression of aromatase, COX-2, and local estrogen production [48]. Estrogen receptor b (ERb) is increased in endometriotic tissue and mediates estradiol-induced COX-2 expression. Also, angiogenesis and migration are strongly linked to IL-6 expression [49]. The expression levels of cytokines and chemokines such as IL-1b, TNF-a, IL-6, and IL-8, secreted by peritoneal macrophages and ectopic endometriotic lesions, were abnormally elevated in peritoneal fluid [50]. IL-1b is a major proinflammatory cytokine released in excess by endometriosis-derived peritoneal macrophages and found in high levels in the peritoneal fluid of endometriosis patients [16]. In 2018, Lousse et al. showed that IL-8 is involved in the pathogenesis of endometriosis, as sex steroids might stimulate the chemokine IL-8 expression in endometrial cells from women with endometriosis [51]. A proinflammatory transcription factor called NF-*κ*B is involved in both physiological and pathological inflammation [52]. Our results in this study are in complete agreement with other studies on the increase of inflammatory factors in endometriosis patients. NF-*κ*B is activated by various inflammatory factors and then stimulates proinflammatory cytokines. NF-*κ*B activator increases inflammation, invasion, angiogenesis, and cell proliferation in endometriosis lesions and peritoneal macrophages in endometriosis patients while suppressing apoptosis [53].

### 4.5. Steroid Signaling

Endometriosis is regarded as an estrogen-dependent disease, and women with this condition display an increased estrogenic expression and activity. Aberrations in the molecular pathways hinder with this hormonal regulation favoring an overproduction of estrogen, prostaglandins, and cytokines which could potentially lead to the onset of endometriosis [54]. We also examined whether there was a relationship between the rate of endometriosis development and estrogen level. According to the results of this study and compatible with previous studies, the expression of estrogen gene in E-MenSCs cells is significantly higher than that in NE-MenSCs [55, 56].

### 4.6. Wnt/*β*-Catenin Signaling

Two stages appear to be essential for the establishment of endometriosis, according to the implantation theory: migration and invasion. These findings indicate that abnormal Wnt/*β*-catenin pathway activation could lead to enhanced migration and invasion of menstrual endometrial cells in endometriosis patients [57]. Cyclin D1 expression was shown to be higher in the secretory phase stromal cells of patients with endometriosis than in the secretory phase stromal cells of healthy women [58]. In this study, a significant increase in *β*-catenin and Cyclin D1 was observed in E-MenSCs compared to NE-MenSCs, which is quite consistent with the observations of previous studies [20, 59, 60]. Cell proliferation, migration, and invasion are all regulated by Wnt/*β*-catenin pathway, which is also involved in the pathophysiology of endometriosis [61].

## 5. Conclusion

In this study, we showed that there are gene expression differences between E-MenSCs and NE-MenSCs. Our results showed that several genes from critical cellular processes including inflammation, apoptosis, migration, and angiogenesis are differentially expressed in E-MenSCs compared with healthy cells. These findings propose that MenSCs could play a key role in the pathogenesis of endometriosis and further support the menstrual blood retrograde theory of endometriosis formation. This could be of great importance in exploiting promising therapeutic targets and new biomarkers for endometriosis treatment and prognosis.

## Figures and Tables

**Table 1:** Specific primers for target genes.

Gene	Sequence	Accession number	Product size (bp)
IL-1*β*	F: TCTTCTTCGACACATGGGATA	NM_000576.3	183
	R: GTACAAAGGACATGGAGAACA		
IL-6	F: GTGTGAAAGCAGCAAAGAGG	NM_000600.5	140
	R: CCTCAAACTCCAAAAGACCA		
IL-8	F: GGAAGGAACCATCTCACTGT	NM_001354840.3	122
	R: GTTCTTTAGCACTCCTTGGC		
COX-2	F: TCAGCCATACAGCAAATCCT	NM_000963.4	205
	R: TTGAAGTGGGTAAGTATGTAGTG		
TNF-*α*	F: GTCTGGGCAGGTCTACTTTGG	MH180383.1	172
	R: GTTCTAAGCTTGGGTTCCGAC		
NF-k*β*	F: GAAGTGCAGAGGAAACGTCAG	NM_001382627.1	147
	R: GAAGCTATACCCTGGACCTGT		
HIF-1*α*	F: GGCGAAGTAAAGAATCTGAAG	NM_181054.3	209
	R: ACCATCCAAGGCTTTCAAATA		
OCT-4	F: GTTCTTCATTCACTAAGGAAGG	NM_001285986.2	101
	R: CAAGAGCATCATTGAACTTCAC		
NANOG	F: ACCTGAAGACGTGTGAAGATG	NM_001355281.2	187
	R: ATTAGGCTCCAACCATACTCC		
SOX-2	F: GGGAAATGGAAGGGGTGCAAAAGAGG	NM_003106.4	151
	R: TTGCGTGAGTGTGGATGGGATTGGTG		
SALL-4	F: GGGCAGCCACATGTCTCAGCA	NM_001318031.2	204
	R: GACATGACGTTCGGGAGCACC		
BAX	F: CGGCAACTTCAACTGGGG	NM_001291430.2	149
	R: TCCAGCCCAACAGCCG		
BCL-2	F: GGTGCCGGTTCAGGTACTCA	NM_000657.3	114
	R: TTGTGGCCTTCTTTGAGTTCG		
MMP-2	F: ACAGTGGATGATGCCTTTGC	NM_004530.6	156
	R: GAGTCCGTCCTTACCGTCAA		
MMP-9	F: GCACCACCACAACATCACCT	NM_004994.3	190
	R: ATACCCGTCTCCGTGCTCC		
Cyclin D1	F: CCCTCGGTGTCCTACTTCA	NM_053056.3	117
	R: GAAGACCTCCTCCTCGCAC		
ER	F: GAGGGGGAATCAAACAGAAAG	NM_001122740.2	201
	R: CTGCTGGATAGAGGCTGAGT		
*β*-Catenin	F: GCGTGGACAATGGCTACTC	NM_001330729.2	203
	R: GCCGCTTTTCTGTCTGGTT		
VEGF	F: TGCTTGCCATTCCCCACTT	NM_001171622.2	195
	R: ACTTTGCCCCTGTCGCTTT		

## Data Availability

The data used to support the findings of this study are available from the corresponding author upon request.
